# Annotations

**Published:** 1900-07-28

**Authors:** 


### Annotations.

### Doctors and the Press.

A question asked in the House of Commons last Friday by Dr. Farquharson raised an interesting point in regard to the right of medical men employed by public bodies to communicate to the Press such observations as they may make and such information as they may pick up in the course of their duty. Dr. Farquharson asked whether it was a fact that civil surgeons employed in military hospitals in South Africa had been required by the Army Medical Department to sign a contract not to divulge in any way what their impressions might be on hospital matters. To this Mr. Wyndham made an answer which was practically in the negative. He read from the form of contract the two paragraphs relating to discipline, the first promising obedience to orders, and the next running as follows:—

"In case I shall complete my service hereunder to your satisfaction in all respects, I shall receive at the end of the said period a gratuity of two months' full pay at the rate thereinbefore specified, but in case I shall in any manner misconduct myself, or shall be (otherwise than through illness or unavoidable accident) unfit in any respect for service hereunder, of which misconduct or unfitness you shall be sole judge, you shall be at liberty from and immediately after such misconduct or unfitness to discharge me from further service hereunder, and thereupon all pay and allowances hereunder shall cease, and I shall not be entitled to any free passage home or gratuity."

And he added, "that is the only contract civil surgeons have to sign, and for my part I cannot discover any foundation for the statement in question." Well, for the life of us we do not see why he should have been so shy, or why on the strength of the mere words of the contract he should deny what was pretty clearly meant. It does not seem to us to be an out-of-the-way thing to say that people are not to write to the newspapers about what they see in the course of their work. We have here close at hand well-known examples, and in regard to the civil surgeons in South Africa, the "misconduct" referred to in the terms of the above contract surely covers anything that would be regarded as misconduct by the officers with whom they mixed and with whom they ranked. Certainly the R.A.M.C. men would not consider themselves at liberty to criticise in the public press the hospitals in which they were employed. The cacoethes scribendi, which has affected some of the consulting surgeons, is another matter, and would have been a source of wonderment had it not been generally understood that they went out to report as well as to consult.

### Sanitary Arrangements for Women Workers.

Women workers ought to be very thankful to the lady inspectors under the Factory and Workshops Act for not having hesitated to deal with a disagreeable subject, and to lay bare the general indecency which is allowed to prevail in many places in regard to the sanitary conveniences afforded to women factory workers, and those men who are employers ought to be very much ashamed of themselves for permitting the continuance in their works of such conditions as are mentioned in the reports made by these lady inspectors. But in addition to the absence of all separate accommodation for women, in some places, and the unsuitability of what is provided, its insufficiency in amount, its indecency, its insanitary condition in others, all of which are contrary to law and capable of remedy, complaint is made that there are large numbers of women workers who only doubtfully come under the Act, if they do so at all, and that for them there is no protection at all in regard to this matter of sanitary conveniences. In the latter class come women clerks and typists, of whom a large number are now employed in all offices. The matter has already become urgent in regard to London, where, in consequence of a curious difference between the wording of the Public Health Acts for London and the country, the ladies working in offices are less protected than their provincial sisters. Everywhere, except in London, "Every building used as a workshop . . . . or where persons are employed . . . . in any trade or business" shall be provided with "proper separate accommodation for each sex," if persons of both sexes "are employed . . . . or in attendance," and it would not be difficult to maintain that typewriting is a "trade or business." In the London Act, unfortunately, the phrase used is, for "every factory, workshop, and workplace." Now is a clerk's office a "workplace" within the meaning of the Act? We are afraid not, and that leaves the lady clerks helpless. But it is high time that the whole question of sanitary accommodation for ladies in city offices should be taken up. Both the Factory and Workshops Act and the Public Health Acts are emphatic with regard to workers in factories and workshops. So far as they are concerned all that is required is to put the law in force, which is being done by the efforts of the lady inspectors; but this being so it is, as the principal lady inspector says, all the more surprising to find that girls who, in the majority of cases, have had a gentler rearing and more refined home atmosphere than the employées in factories, should lack in this particular the protection of the law.

### Where to Vaccinate.

There can be very little doubt that the spot on the outside of the arm, where vaccination is usually performed, has been chosen from motives of convenience rather than æsthetics, and with but small reference to the feelings of the little patients in after life. So far as convenience is concerned nothing could be better; and when the good time comes when to be unvaccinated will be considered a disgrace, no doubt every young lady will be proud to carry her scars open to public view. In the meantime they don't like it, and many attempts have been made to find a better place. On the whole, the most popular substitute seems to be the leg. The objection to using the leg as a site for vaccination arises chiefly from the difficulty which is found by some mothers in keeping the part dry and clean; but with modern methods of protected vaccination this may not prove so important as it was when all vaccinations were left bare. Take it altogether, however, the most generally convenient place will probably be found to be on the left flank, over the lower ribs; in fact, just above where the child's napkin comes. If babies were reasonably dressed, that would be a spot on which but little friction need ever fall, on which it would be easy to apply a protective dressing, and on which the necessary area of cicatrix would not become a deformity. Moreover, much of the trouble which sometimes follows vaccination arises from the area of vesication required, and its being crowded on the small extent of surface offered by the arm. But with a larger area to work upon the vesicles might be further apart, and it is not impossible that much secondary inflammation might be saved. All, however, depends upon how the baby is dressed. So long as the attempt is made to make an infant's clothes fit, it is no use vaccinating just where they are sure to rub, and the choice probably lies between arm and leg—arms for boys and legs for girls.

